# Identifying Parameters for Defining “Essentially Derived Varieties” of Maize Inbred Lines Using High-Throughput Genome-Wide SNP Markers

**DOI:** 10.3390/plants11151909

**Published:** 2022-07-23

**Authors:** Yuanyuan Yan, Shanqiu Sun, Ruixia Xing, Haiyang Jiang, Beijiu Cheng

**Affiliations:** 1 School of Life Sciences, Anhui Agricultural University, Hefei 230036, China; yanyuanyuan0310@163.com (Y.Y.); sunsq8903@163.com (S.S.); 17853555073@163.com (R.X.); hyjiang@ahau.edu.cn (H.J.); 2 Institute of Crop Science, Chinese Academy of Agricultural Sciences, Beijing 100081, China

**Keywords:** high-throughput genotyping, essentially derived variety, residual heterozygosity, genetic drift, SNP chip array

## Abstract

Well-developed maize reference genomes and genotyping technology along with fast decreasing detection costs have enabled the chance of shifting essentially derived varieties (EDV) identification to high-throughput SNP genotyping technology. However, attempts of using high-throughput technologies such as SNP array on EDV identification and the essential baseline parameters such as genetic homozygosity and/or stability in EDV practices have not been characterized. Here, we selected 28 accessions of 21 classical maize inbreds, which definitely form a pedigree network from initial founders to derivatives that had made huge contribution to corn production, to demonstrate these fundamental analyses. Our data showed that average residual heterozygosity (RH) rate of these 28 accessions across genome was about 1.03%. However, the RH rate of some accessions was higher than 3%. In addition, some inbreds were found to have an overall RH rate lower than 2% but over 8% level at certain chromosomes. Genetic drift (GD) between two accessions from different years or breeding programs varied from 0.13% to 13.16%. Accessions with low GD level showed cluster distribution pattern and compared with RH distributions indicated that RH was not the only resource of GD. Both RH and GD data suggested that genetic purity analysis is an essential procedure before determining EDV. Eleven derivative lines were characterized with regard to their genome compositions and were inferred as their breeding histories. The backcross, bi-parental recycling, and mutation breeding records could be identified. The data provide insights of underlining fundamental parameters for defining EDV threshold and the results demonstrate the EDV identification process.

## 1. Introduction 

The increasing population and consumption in the world are presenting unprecedented challenges to agriculture to meet food security and sustainability needs [1]. Meanwhile, the adoption of developing agricultural techniques [2] is central to minimize extensive losses due to abiotic stresses under global climate change [3]. Crop breeding is one of the key routes through which increased production, efficiency, and sustainability can be delivered to address these challenges [4]. De novo breeding, which originated from primitive germplasm to create new varieties, is a long effort and cost-extensive process. Comparably, there should be faster, much more efficient, and relatively easier to breed varieties from the existing elite varieties. Therefore, an effective intellectual property protection for the initial variety needs a balance between to incentivize original innovation and to promote breeding efficiency.

To fulfill the mission of plant variety intellectual property protection, the International Convention for the Protection of New Varieties of Plants (UPOV Convention) was established and adopted in Paris in 1961, and it was revised in 1972, 1978, and 1991 (UPOV Convention. Available online: https://www.upov.int/ (accessed on 22th July 2022). Beyond the 1961, 1972, and 1978 UPOV Conventions, the 1991 UPOV Convention (hereinafter 1991 UPOV) had introduced and adopted “essentially derived varieties” (EDV), which expand the scope of a breeder’s right to derived variety that was selected on the basis of a minor difference, a mutation, a genetic modification, a backcross, or a selection. The 1991 UPOV was accepted by the mainstream as a rule to achieve the balance of variety rights protection and research efficiency; though, it is still being debated in some large developing countries including China. In the contemporary era of rapid technology development, it is urgent to protect the right of initial varieties due to new advent technologies, such as transgene, physical, chemical, and biological mutagenesis, which may introduce traits or mutations into a given variety. For instance, genome editing has enabled researchers to precisely modify a desirable genotype of a given gene in crops [5]. Taking maize as an example, we had edited a number of important agronomic traits, such as waxy [6,7], super-sweet [8], plant architecture [9], fertility, and herbicide resistance [10], which can quickly improve the target traits of the recipient varieties. Therefore, the protection of initial variety rights is related to the sustainability of agricultural research and development and will eventually be recognized by governments all over the world.

The 1991 UPOV has been used in pioneer and major seed industries for about 30 years. However, determination of germplasm ownership is unwieldy and limits progress [11] of the 1991 UPOV extension. Melchinger et al. [12] assessed similarity for RFLPs (restriction fragment length polymorphisms) among related and unrelated maize inbreds. Later on, AFLP (amplified fragment length polymorphism) and SSR (simple sequence repeats) markers were recommended and used for this purpose in maize [11,13,14,15,16,17]. The RFLP, AFLP, and SSR molecular markers are time and cost intensive experiments that limited the numbers of markers employed in practice. The biggest number of 285 SSR markers had been reported in EDV evaluation in maize [18]. As the cost of single nucleotide polymorphism (SNP) detection continued to decline, a set of 3072 SNPs having even genomic coverage was recommended to provide robust, precise, and discriminatory capacity along with providing good comparison with SSR marker [19]. Maize reference genomes along with re-sequencing studies [20,21,22,23] had provided a wealth of a hundred million SNP across the genomes. Due to the refined identification of genome representations by genome-wide high-throughput SNP data, it is becoming a trend to use high-throughput SNP technology to define the genome composition of varieties. Actually, the principle of new variety on distinctness, uniformity and stability (DUS) based on trait phenotypes should also be applicable to any protocols adopting molecular markers in genome. Firstly, an ideal parental inbred line is presumed to have 100% genetic homozygosity corresponding to uniformity of DUS; however, the actual genome of a given inbred harbors RH, mutation, or a combination of both [24]. Secondly, to what extent the genetic stability or genetic drift will happen among different accessions due to RH and mutations needs to be characterized. This point corresponds to the stability of DUS. Thirdly, based on two above-mentioned points, how to use high-throughput data to analyze the parental genome composition also needs to establish a quick and simple analysis process of its distinctness. In short, fundamental parameters and standards for EDV determination using high-throughput genome-wide SNP markers should be set-up.

To address these concerns, we selected some classical maize inbred lines with clear breeder’s empirical pedigree (Supplementary Figure S1) as the test materials. They had been largely applied to maize breeding and further made huge contributions to corn production in China. These accessions of the inbred lines, which had reliable seed bank records, had been analyzed with regard to the RH and GD and then been analyzed with regard to their genome contributions from initial inbred founders. The data and process provided some insight of underlining fundamental parameters for defining maize parental line EDVs and could be extended to other agriculture species using high-throughput genome data.

## 2. Materials and Methods

### 2.1. Maize Inbred Lines and Accessions

A total of 21 maize inbred lines with 28 seed bank accessions were used in the present study (Supplementary Table S1). All inbred lines were highly selected based on the importance of the germplasm, a clear breeder’s empirical pedigree (Supplementary Figure S1) with historically verified breeding programs along with high seed purity based on senior breeders’ experiences. Incomplete official statistics data showed that these inbred lines had bred at least 131 single-cross maize hybrids with total growing area over 122.44 Mha of field corn production in China. The official data were publicly available from the China Seed Industry Data Platform (China Seed Industry Data Platform. Available online: http://202.127.42.47:6006/home [accessed on 22th July 2022]). Items with unavailable data entries indicate that the actual application area may not met the statistical data inclusion criteria. All maize inbred lines were generously gifted from Maize Research Institute, Jilin Academy of Agricultural Sciences, which is the one of the most important research institutes and contributed major germplasm and varieties for China temperate maize.

### 2.2. Sampling and Genomic DNA Extraction 

About 100 seeds of each accession were germinated cultured in sand until 2-week-old seedlings to verify the seed purity before sampling. A bulk of 15 plants for each accession were sampled for the young seedling leaves to isolate genomic DNA using a Plant Genomic DNA Kit (DP350-03, Tiangen Biotech Co., Ltd., Beijing, China), according to the manufacturer’s protocols.

### 2.3. Genome-Wide High-Throughput SNP Genotyping, Clean Data Filtering, and Generating

Genome-wide high-throughput SNP genotyping was scored using an Axiom^®^ Maize56K SNP Array, which contains 56,000 SNPs. The hybridization signals were detected using a GeneChip™ Scanner 3000 7G (00-0210, Thermo Fisher Scientific Co., Ltd., California, US) to obtain raw CEL files. The CEL files were processed using the Axiom Analysis Suite 5.0.1 (Thermo Fisher Scientific Co., Ltd., San Jose, CA, US). The dish quality of samples was > 0.82. The SNPs in this array were distributed evenly across maize 10 chromosomes. The SNP array was designed based on huge, previewed SNPs and reliably high polymorphic loci and probes among Chinese commonly used maize germplasms. The genotyping and raw data filtering were obtained from a service provided by China Golden Marker Biotech. Co., Ltd. (Beijing, China). The physical haplotype map was constructed according to B73 RefGen v4. The missing data were removed by pairwise analysis between accessions. The call rate criterion of SNP was set at 97%. The SNPs, which were categorized into PolyHighResolution, MonoHighResolution, or NoMinorHom, were used in the present study. The high stringent, filtered SNP data were generated for the specific analysis (Supplementary Table S3). Briefly, the loci with missing data of each accession were removed for RH analysis. The loci with missing data among different accessions were all removed for GD analysis on each inbred. The polymorphic SNP loci between founder lines of the derived line were applied into EDV analysis. 

### 2.4. RH Analysis

High stringent, filtered SNP genotyping data without missing data were used in analysis. The RH rate for each accession was calculated for each chromosome and for the whole genome. The rate is the number of heterozygous loci divided by total numbers of the applied loci, expressed as a percentage. The further RH abundance analysis along each of the 10 chromosomes across the 28 accessions was performed by applying a sliding window of 50 SNP loci. The total number of RH loci among the 28 accessions in this window were plotted as a dot for the Y variables. The dots for representing the windows on pericentromeric regions of each chromosome were shown in red.

### 2.5. GD Analysis

Here, the accessions of same inbreds shared the same seed origins. GD here refers to the SNP loci variations owing to the chances of sampling distortion, fitness selection from inbreds with low level heterozygosity, and natural mutations. Five lines, Zi330, Ji853, 444, Jin03, and DH02, with two different accessions and one line of HZ4 with triple accessions were used to analyze the genetic drift between different accessions of the same year or accessions between depositions of different years. A SNP locus with different genotypes between two accessions is a putative genetic drift locus. Percentages were calculated for all putative loci and maps were generated using the ggplot2 package in R [25].

### 2.6. Analysis of Genome Compositions from Parental Founder Lines

The genome composition of an EDV or a partially derived inbred was analyzed from two founder lines based on empirical pedigree information. The SNP loci harboring missing data and RH were removed before analysis. Then, the polymorphic SNPs between two founder lines were generated to analyze the representation of the genomic origin of either founder. The defined genome origins were plotted throughout genome with two different colors. The loci with heterozygous genotypes between two founder lines were shown in a third color. 

### 2.7. Generating Genome-Wide Plot and Profiles Output

Ten chromosome plots of whole genome were conducted and outputted using the ggplot2 package in R [25]. The functions, including ggplot, geom_point, scale_color_manual, draw pairwise venn, and draw triple venn, were used to produce profiles. The physical position of SNPs was used as the X variable. The SNP loci on chromosome were used as the Y variable. As for the RH output, the number of accessions out of the total 28 accessions at each found heterozygosity SNP locus were plotted as the Y variable. As for the GD analysis, SNP loci with different genotypes between two accessions of the same lines were mapped to the color variable. As for genome composition output, the derived line’s SNP loci defined from different founders were mapped to the color variable. 

## 3. Results

### 3.1. Evaluation of Genome-Wide RH Landscape among These Classical Inbred Lines

**Figure 1:**
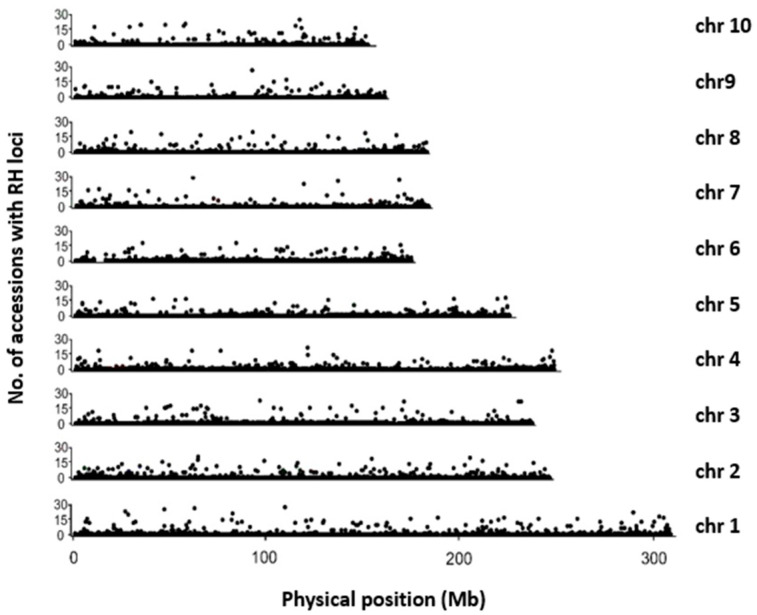
Genome distribution pattern of found RH SNPs among 28 diverse inbred line accessions.

**Figure 2:**
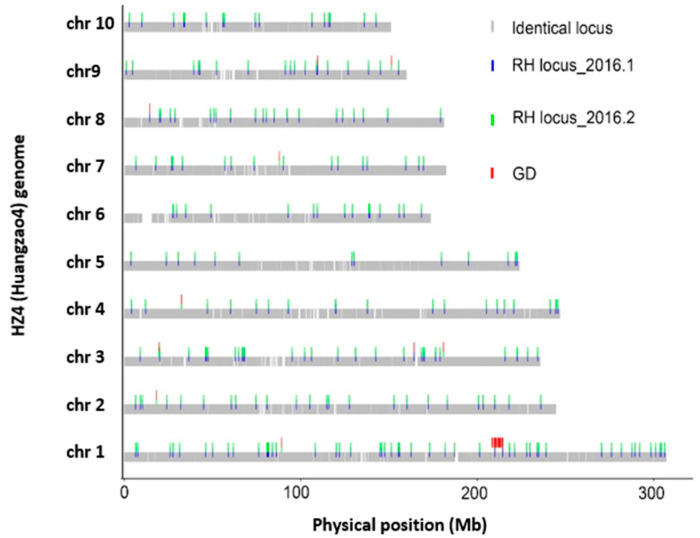
Region distribution of residual heterozygosity (RH) and genetic drifts (GD) between two HZ4 accessions of year 2016 across whole physical genome. An identical locus is a homozygous SNP and is the same in the two HZ4 accessions. A GD locus is a homozygous SNP but is different between the two HZ4 accessions.

One of the primary goals of this study is to evaluate the genome-wide RH landscape of these classical inbred lines. The presented data on RH rate and distribution will provide a reference baseline for defining the EDV threshold. Our data showed that the overall RH rate of these 28 accessions was about 1.03% (Table 1). Under the current stringent SNP filtering parameter, the RH rate of most accessions was lower than 1% within chromosomes or at whole-genome scale. RH loci of 28 accessions were distributed across all over the genome (Figure 1). Some SNP loci were identified RH among the most tested accessions. These data suggested that the genetic purity of the tested classical inbred lines from breeders was good since the scored RH rate was lower than routine level of 3%, which had been commonly reported from genetic studies [26]. However, in the 444_2016 accession, the average RH rate was quite low but with an 8.37% RH on chromosome 5 (Table 1). RH rates of two accessions, DH02_2011 and S2024, reached 4.96% and 3.23%, respectively (Table 1). Moreover, for a specific chromosome such as on chromosome 4 of DH02_2011, the RH rate was as high as 17.74%. On S2024 chromosome 3, the RH rate was as high as 13.61%. Furthermore, in order to analyze the RH distribution pattern along each chromosome, we performed the analysis on sliding windows of 50 SNP loci. In general, the RH abundance of centromere segments was significantly higher than average, which is consistent with the previous report [26]. RH levels of sliding windows overlapped with centromere were generally higher than average rate (Supplementary Figure S2). Interestingly, some low recombination regions [27] also showed higher RH rates (Supplementary Figure S2). Besides, the genome plotted RH loci between accessions of the same lines showed the same physical genome locations (Figure 2, Supplementary Figures S3 and S4). This result indicated that our scored SNP data were reliable since these genotyping data should not have resulted from a false positive during chip hybridization and data processing process.

In summary, our data indicated that the RH rate among most inbred accessions at long-term preservation was about 1.03%. However, there was possibility of some parental lines harbor RH rate as high as over 15% on particular chromosomes or regions. The RH was abundantly distributed throughout the whole genome but showed higher rates at centromeres and some possible recombination cold spot regions. 

### 3.2. Determining GD Levels That Are Essential for Parental Inbred Genome Stability

Six pairs of accessions of the same inbred lines sharing the same seed origins were used in GD analysis (Table 2). The results of three pairs showed that the proportion of GD were quite low, reaching to 0.13%, 0.49%, and 0.44%. However, another three pairs scored substantial GD ratios up to 6.22%, 5.96%, and 13.16%. To further analyze the GD distribution pattern and its correlation with RH, genome-wide RH and GD were simultaneously plotted on genomic physical maps (Figure 2, Supplementary Figures S3, S4, and S5). GD-distributed loci intensively gathered on the genome, and these cluster regions did not overlap with RH cluster loci among low RH rate accessions (Figure 2, Supplementary Figures S3 and S4). For instance, a big GD fragment was found on chromosome 1 with a length of 6.11 Mb between HZ4_2016.1 and HZ4_2016.2, where there were no RH clusters (Figure 2). Surprisingly, inbred 444 and Jin03 scored high GD rates of 6.22% and 5.96% between the 2012 and 2016 accessions (Table 2) but with low RH rates in either accession (Table 1). More evidence for big GD segments could be found on chromosomes 1, 2, and 4 of Zi330 (Supplementary Figure S3) and chromosomes 2 and 5 of Ji853 (Supplementary Figure S4). The results indicated that chromosomal mutations such as jumping transposons might be another important genetic cause of GD in low RH accessions (Figure 2, Supplementary Figures S3 and S4). Therefore, it is suggested that GD analysis is also an essential concern before determining EDV when using high-throughput genome-wide SNP genotyping. 

### 3.3. Cases of EDV Determination by Using High-Throughput Genome-Wide SNP Genotyping

**Figure 3:**
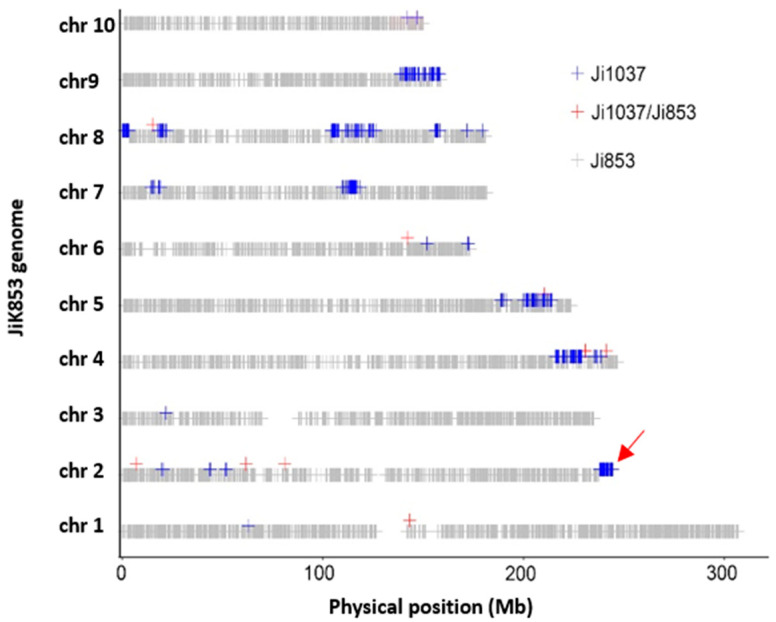
Characterization of both donor (Ji1037) and recurrent (Ji853) parental genome contributions to JiK853, which was essentially derived varieties of Ji853 through backcross breeding. The red arrow indicates the genome position of the head smut resistance introgression from Ji1037.

To demonstrate EDV determination application, we selected some well-known EDVs and their founder lines based on empirical pedigree (Table 3) to determine the genome compositions. Eleven derivative lines were characterized as to their genome compositions and were inferred as to their breeding crossing histories (Table 3). For the JiK853 and JiK287 instances, these two lines are EDVs of Ji853 and Ji287 for head smut tolerance breeding using trait introgression from Ji1037, a disease tolerant donor. The initial founder inbred, Ji853, contributed about 96.04%, while the trait donor line, Ji1037, contributed 3.84% of the JiK853 genome with a small proportion of just 0.12% with unknown origins (Table 3). The initial parental founder inbred Si287 contributed 88.83% of the JiK287 genome. The genome plotted profile of JiK853 clearly showed the head-smut-resistance locus surrounding *ZmWAK* [28], which locates at bin 2.09 is from donor parent of Ji1037 (Figure 3). On JiK853 chromosomes 4, 5, 7, 8, and 9, there were about nine big introgression segments along with a number of very small segments from Ji1037. Similarly, JiK287 was also identified using desirable trait introgression along with some large or small genome segmentations from Ji1037 (Supplementary Figure S6).

To further explore the application scenarios of high-throughput genome-wide SNP technology in this field, we attempted to analyze the EDVs, of which we know only one of founder lines. The A619Ht, which is an EDV inbred of A619 without information on northern leaf blight (*Ht*) donor, was verified with regard to its EDV identification (Supplementary Figure S7). Based on IBM2 2008 Neighbors 2 and B73 RefGen v4, the tightly linked marker umc150b with *Ht1* was located approximately at 218,321,093 on chromosome 2 [29], and a large fragment of 6.45 Mb defined by the SNP AX-86257895 (217,561,726) and the SNP AX-86326665 (224,010,103) on was found in A619Ht on Bin 2.08 but not from A619. An additional example was JiV057, which was recorded as the 444-mutation line based on empirical pedigree record. However, our data on JiV057 suggested that it was substantially derived from 444 but with 14.98% of unknown genome origins rather than a 444-mutation line (Table 3, Supplementary Figure S8). Our analysis showed EDV from backcross, bi-parental recycling, and mutation breeding could be identified using high-throughput SNP genotyping.

## 4. Discussions 

### 4.1. Suggestions on EDV by Using High-Throughput Genome-Wide SNPs

Regarding adoption of whole-genome high-throughput SNP method, the EDV threshold is obviously a core concern, but this threshold is not just a technical issue. One of the most important objectives of the 1991 UPOV Convention is the introduction of the EDV concept extending the breeder’s right to a variety that was selected basis on a minor difference, a mutation, a genetic modification, a backcross, a selection within a variety, and so on. In principle, the EDV concept is also expected to protect the breeding of new varieties from an initial parental line and/or hybrids. However, both the complexity of breeding and technical reasons makes it difficult to establish a simple and effective technical solution to meet clearly defined variety rights boundaries. On the one hand, concerning breeding practice, Troyer et al. [11] suggested raising dependency standards for parenting inbreds to 90% or more and shortening the EDV right to the independent variety to 5 years. The logic behind this suggestion is at least partially rational and scientific because the modern maize parental line breeding relies on recycling breeding, which use bi-parental lines or few lines as founder materials for selecting elite offspring lines. Overclaiming of EDV rights of initial lines based on the 1991 UPOV principle would be an important obstacle to maize breeding. On the other hand, concerning EDV identification itself, it is inapplicable to establish a general standard among the methods due to the different technical characteristics. For example, a comparison between AFLP and SSR data had been conducted on EDV identification due to the differences on genetic distance characterization and marker systems with different degree of polymorphism [13]. Based on the similar considerations, the International Seed Federation (ISF) issued a guideline that a threshold of 91% was set on shifting the burden proof to the breeder of the putative EDV [30]. The high-throughput SNP and sequencing technology had been promoted due to rapid technology development [31]. In this regard, the current study provided fundamental parameters of RH and GD underlining for defining threshold of maize EDVs and also determined the genome-wide parental contribution on putative EDVs. 

### 4.2. RH and GD Analysis as a Fundamental Parameter before EDV Characterization 

The genetic homozygosity of the ideal parental inbred line is 100%. This feature is beneficial for inbred breeding practices due to expected genetic transmission and selective advantage [32]. However, this is not realistic for breeding and seed industrial practices. Theoretically, only inbreed crossing will result in infinitely close to genome homozygosity, but the fitness selections across multiple rounds of selfing crosses unconsciously resist inbreeding depression and result in preserved heterozygosity due to the deleterious mutations with different alleles in repulsion or heterozygote advantage [33]. Therefore, RH presented in maize parental inbreds of hybrids is inevitable. Our data indicated that most of the analyzed lines harbored RH level under 1%. In most cases, the parental lines of seed industrial inbreds would not encounter RH issues when applying large scale SNPs. However, some lines were found quite with high levels of RH (Supplementary Figure S5); some genome regions of some inbreds harbor substantial RH level at specific regions (Supplementary Figure S8 and Table 1), though, the overall RH level was low. These data suggested that RH or heterozygosity analysis is an essential procedure before determining EDV when using high-throughput genome-wide SNP genotyping. 

Another aim in the present study is to analyze the extent of genetic variation caused by GD. This issue is also an essential parameter that is highly correlated with stability of parental inbreds and, therefore, hybrids in maize. Advances in both high-throughput genotyping and the maize reference genomes now enable the fine DNA segmentation tracing across the whole genome. Some accessions of the same lines and sharing the same seed origins were analyzed. Our data showed the GD may result from RH and some genome instability elements. Since RH is important resource of GD, overlapping GD and RH on chromosomes 4, 6, and 8 of DH02 can be well-explained by this hypothesis (Table 1 and Table 2, Supplementary Figure S5). There were also some big regions on chromosomes 3 and 9 with high levels of GD chromosomal variation, supposedly from pollinating containments (Supplementary Figure S5). To sum up, GD presented on genome at a low level but was distributed in clusters that may have resulted from RH or chromosomal mutations along with possible pollination containments. Both RH and GD analyses in this study suggested that genetic purity analysis across whole genome is an essential procedure before determining EDV using high-throughput genome-wide SNP genotyping. In addition, the seed purity standards and identification methods need to be improved, since the lines with abnormally high RH and GD levels had been tested, satisfying current trait-based DUS trait test in this study. In another sense, an improved seed purity quality will also be beneficial for marketing life due to lower RH and GD.

### 4.3. Perspective of the Application Scenarios of EDV Identification Using High-Throughput Genome-Wide SNP Technology

With the developed maize reference genomes [21,22,23] and the ever developing of the SNP detection technology, the costs of genome-wide high-throughput SNP have fallen rapidly. Maize EDV identifications shifting to high-throughput SNP technologies are trending. In this study, we had made some preliminary attempts to apply high-throughput SNP into some specific application scenarios of maize EDV identification on parental inbreds. For instance, we could clarify the specific genomic contribution on EDV inbred from original founder lines or initial varieties (Table 1, Figure 3, Supplementary Figures S6 and S7). For an inbred line where we know only one major contributor of the initial founder line with such an incomplete pedigree record such as JiV057, the genomic contribution of this initial founder of 444 was clearly characterized (Table 1, Supplementary Figure S8). Obviously, new technology has laid a good technical foundation for this application, and new standards based on new technologies will be more objective and scientific, further demonstrating fairness and justice in EDV law enforcement in seed industry applications. Looking to the future, it is worth noting that further application scenarios are yet to be established for the analysis technology system, for example, how to determine the genome compositions from a large scale of initial varieties, how to determine both EDV right of hybrid and parental lines from a single-cross hybrids, etc. In short, high-throughput genotyping methods will bring new developments to EDV identification.

## Figures and Tables

**Table 1:** Estimation of overall and single-chromosomal genome residual heterozygosity levels among maize inbred accessions using genome-wide SNP genotypes.

Inbred Accessions	Percentage of Heterozygous SNP within Chromosomes (%)										Aver. (%)
	chr.1	chr.2	chr.3	chr.4	chr.5	chr.6	chr.7	chr.8	chr.9	chr.10	
HZ4_2009	0.84	0.84	0.74	0.49	0.40	0.49	0.58	0.90	0.65	0.80	0.67
HZ4_2016.1	0.91	0.60	0.77	0.52	0.37	0.59	0.54	0.68	0.73	0.80	0.65
HZ4_2016.2	0.91	0.60	0.82	0.55	0.40	0.59	0.61	0.64	0.69	0.76	0.66
444_2012	0.82	0.77	0.89	0.55	0.50	0.70	0.61	0.48	0.62	1.10	0.70
444_2016	0.87	1.44	1.16	0.55	8.37	2.34	0.58	0.77	2.19	1.01	1.93
Jin03_2012	0.82	0.75	0.62	0.81	0.77	0.70	0.72	0.84	1.04	1.14	0.82
Jin03_2016	0.68	1.18	0.52	0.75	0.60	0.66	0.65	0.68	0.81	1.05	0.76
DH02_2011	1.36	1.32	3.79	17.74	1.37	11.26	2.25	6.19	1.73	2.61	4.96
DH02_2016	0.72	0.72	0.82	0.73	0.50	0.59	0.95	0.87	0.50	0.84	0.72
Zi330_2015	0.72	1.06	0.54	0.78	0.60	0.49	0.48	0.55	0.38	0.97	0.66
Zi330_2017	0.72	1.06	0.54	0.78	0.57	0.52	0.44	0.55	0.38	0.97	0.65
Ji853_2016.1	0.80	0.65	0.64	0.70	0.60	0.59	0.82	0.71	0.69	0.80	0.70
Ji853_2016.2	0.80	0.58	0.64	0.73	0.65	0.59	0.82	0.68	0.62	0.84	0.70
JiK853	0.77	0.67	0.64	0.94	0.62	0.59	0.75	0.74	0.69	0.80	0.72
A619	0.66	0.89	3.56	2.75	0.70	0.80	1.87	0.55	0.42	1.14	1.33
A619Ht	0.70	0.89	0.47	0.88	0.72	0.87	0.44	0.61	0.46	1.22	0.73
B467	0.82	0.79	0.62	0.81	0.55	0.52	0.75	0.74	0.73	1.26	0.76
JiV057	0.79	0.89	0.89	0.65	0.55	0.59	0.65	0.58	0.69	1.18	0.75
Si287	0.92	1.30	0.77	0.73	0.67	0.63	0.61	0.61	1.04	0.97	0.83
J9206	0.79	0.72	0.79	0.55	0.80	0.80	0.78	0.97	0.77	0.97	0.79
Ji1037	0.94	0.94	0.97	0.68	0.87	0.80	0.92	0.77	0.58	0.93	0.84
JiK287	0.72	1.08	0.69	0.44	0.82	0.49	0.65	0.61	0.96	1.01	0.75
L269	0.66	0.63	0.67	0.55	0.60	0.77	0.78	0.58	0.38	0.80	0.64
S1014	0.59	0.89	0.77	0.94	0.55	0.63	0.82	0.58	0.96	0.84	0.76
PHP02	1.15	0.70	0.72	0.52	0.95	0.45	0.68	0.64	0.73	0.55	0.71
S2024	0.80	1.32	13.61	7.30	0.72	1.99	2.21	2.06	1.23	1.10	3.23
Si273	0.86	0.79	0.77	0.52	0.42	0.70	0.54	0.71	0.50	0.97	0.68
JiA3301	0.80	1.01	0.87	0.49	0.62	0.70	0.54	0.55	0.81	1.05	0.74
Aver. chr.	0.82	0.90	1.40	1.59	0.92	1.12	0.82	0.92	0.79	1.02	
								Overall average			1.03

**Table 2:** Evaluation of the genetic drift between two accessions of inbreds using genome-wide SNP genotypes.

Comparing Inbreds between 2 Accessions	HZ4_2016.1 vs. 2016.2		Zi330_2015 vs. _2017		Ji853_2016.1 vs. 2016.2		444_2012 vs. 2016		Jin03_2012 vs. 2016		DH02_2011 vs. 2016	
	No.	%	No.	%	No.	%	No.	%	No.	%	No.	%
**chr.1**	37	0.65	53	0.93	2	0.04	482	8.47	388	6.81	561	9.86
**chr.2**	1	0.02	49	1.18	112	2.70	593	14.37	573	13.87	345	8.34
**chr.3**	3	0.07	2	0.05	0	0.00	184	4.58	13	0.32	999	24.88
**chr.4**	1	0.03	36	0.94	2	0.05	29	0.76	74	1.93	669	17.51
**chr.5**	0	0.00	1	0.02	35	0.87	715	17.98	74	1.86	242	6.05
**chr.6**	0	0.00	13	0.46	1	0.04	59	2.08	284	10.00	413	14.53
**chr.7**	1	0.03	1	0.03	5	0.17	3	0.10	11	0.38	179	6.12
**chr.8**	1	0.03	0	0.00	0	0.00	59	1.91	544	17.70	314	10.20
**chr.9**	2	0.08	0	0.00	1	0.04	73	2.82	5	0.19	868	33.66
**chr.10**	0	0.00	18	0.76	0	0.00	7	0.30	146	6.19	76	3.21
**Total**	46	0.13	173	0.49	158	0.44	2204	6.22	2112	5.96	4666	13.16

**Table 3:** The inferred genome composition of derivative line based on genome-wide SNP data and comparison between their bi-parental founders from the empirical pedigree.

Derivative Lines	Founder Line 1		Founder Line 2		Unknown (%)	Inferred Pedigree (Recurrent Parent in Bold)
	Name	%	Name	%		
**444**	HZ4	60.79	A619ht	37.01	2.20	HZ4×A619ht
**Ji853**	HZ4	79.75	Zi330	20.09	0.16	HZ4×Zi330 BC1
**JiK853**	Ji853	96.04	Ji1037	3.84	0.12	Ji853 *×Ji1037 BC3
**S2024**	Si287	79.97	PHP02	19.76	0.27	Si287 *×PHP02 BC1
**J9206**	444	48.88	DH02	50.81	0.31	444×DH02
**JiK287**	Si287	88.83	Ji1037	11.03	0.14	Si287 *×Ji1037 BC2
**S1014**	Si287	59.39	L269	40.45	0.16	Si287×L269
**S2024**	Si287	39.79	PHP02	52.48	7.73	Si287×PHP02
**JiA3301**	Si287	35.34	Si273	64.54	0.12	Si287×Si273 or Si287 *×**Si273**BC1
**JiV057**	444	85.02	Unknown	Unknown	14.98	444 *×Unknown BC2
**B467**	444	74.11	434	NA	25.89	444 *×434BC1

Note: *, The inbred name in bold indicated the recurrent parental line of BC (backcrossing) breeding.

## Data Availability

Not applicable.

## Supplementary Materials

The following supporting information can be downloaded at: https://www.mdpi.com/article/10.3390/plants11151909/s1, Figure S1: Empirical pedigree networks of the analyzing maize inbreds; Figure S2: Sliding window analysis of the RH abundance distribution along each 10 chromosomes of 28 accessions; Figure S3: Comparison of residual heterozygotes (RH) and genetic drifts (GD) between 2 accessions of Zi330, one of the classic maize founder lines of wide applied germplasm; Figure S4: Comparison of residual heterozygotes (RH) and genetic drifts (GD) between 2 accessions of Ji853, one of the classic maize founder lines of wide applied germplasm; Figure S5: Comparison of residue heterozygotes (RH) and genetic drifts (GD) between 2 accessions of DH02, one of the classic maize founder lines of wide applied germplasm; Figure S6: Characterization of both donor (Ji1037) and recurrent (Si287) parental genome contributions to JiK287, which was essentially derived varieties of Si287 through backcross breeding; Figure S7: EDV analysis of A619Ht genome from A619 (homologous loci) and unknown origins (heterozygous loci); Figure S8: Identification of JiV057 genome, which was essentially derived from 444 with around 14.98% genome introgression from unknown origins but not 444 with a single or multiple loci mutations. Table S1: Accessions of maize inbreds used in this study; Table S2: The incomplete offical data on hybrids and their growing area as analyzed inbred as parental lines; Table S3: The number of SNP applied into analysis.

## Abbreviations

AFLP	amplified fragment length polymorphism
DUS	distinctness, uniformity and stability
EDV	essentially-derived-varieties
GD	genetic drift
ISF	the International Seed Federation
RFLP	restriction fragment length polymorphism
RH	residual heterozygosity
SNP	single nucleotide polymorphism
SSR	simple sequence repeat
UPOV Convention	the International Convention for the Protection of New Varieties of Plants
